# Transcriptomic Signatures in TP53 Positive and Negative Tumor Samples in NSCLC

**DOI:** 10.2174/0115665232357300250805021330

**Published:** 2025-08-08

**Authors:** Miao Xie, Baoguang Liu, Ziyi Chen, Tongtong Cao, Xiaoyan Zhang

**Affiliations:** 1 College of Traditional Chinese Medicine, Henan University of Chinese Medicine, Zhengzhou, Henan, China;; 2 Department of Chinese Medical Sciences, Henan University of Chinese Medicine, Zhengzhou, Henan, China

**Keywords:** TP53-associated transcriptomic signature, lung cancer, NSCLC, survival, gene expression, immune cell

## Abstract

**Introduction:**

Lung cancer, specifically non-small cell lung cancer (NSCLC), is a leading cause of cancer-related mortality worldwide. TP53, a crucial tumor suppressor gene, is often mutated in various cancers, including lung cancer. This study focuses on the differences in transcriptomic profiles between TP53-mutated (TP53+) and TP53-wildtype (TP53-) NSCLC tumor samples, aiming to develop a gene signature that can predict overall survival and immune response, particularly in the context of immunotherapy. It aims to identify differentially expressed genes (DEGs) associated with TP53 status in non-small cell lung cancer and develop a gene signature that can predict overall survival and immune response.

**Methods:**

Gene expression profiles from TP53-positive and TP53-negative NSCLC tumor samples were analyzed. Data were sourced from the GEO database (GSE8569, n = 69) and the TCGA database (n = 1026). Differential expression analysis was conducted to identify DEGs, which were further analyzed using LASSO regression to develop a prognostic gene signature. Quantitative PCR (qPCR) was performed to validate the expression of selected genes.

**Results:**

A total of 535 DEGs (168 up-regulated, 367 down-regulated) were identified in TP53+ samples. Further analysis with TCGA data narrowed this down to 29 genes, from which 12 were identified as prognostic features using LASSO analysis. This 12-gene signature effectively stratified patients into low- and high-risk groups for overall survival. Differences in immune cell infiltration and immune pathway activity were significant between these groups, indicating the potential of the gene signature to predict immune response. Among the genes analyzed, BMP2, LPXN, IER3, ANLN, TNNT1, OGT, KRT8, BARX2, PRC1, and SNX30 showed statistically significant differences in qPCR results.

**Discussion:**

The 12-gene signature demonstrates robust predictive capability for survival outcomes and immune response patterns in NSCLC patients, suggesting its potential clinical utility in precision oncology. The observed correlation between TP53 mutation status and immune microenvironment alterations provides valuable insights into the mechanistic basis of immunotherapy resistance and response.

**Conclusion:**

This study identifies a TP53-associated transcriptomic signature that is significantly associated with overall survival in lung cancer patients. The gene signature also correlates with differences in immune cell infiltration patterns between risk groups, offering potential insights into the tumor immune microenvironment. These findings may contribute to future efforts to stratify patients and guide immunotherapy decisions, pending further experimental validation.

## 1 INTRODUCTION

Lung cancer remains a formidable adversary in the arena of public health, bearing the unfortunate distinction of being the most common cause of cancer-related mortality globally [1]. This dire status is underscored by the staggering statistics that reflect its impact: it is estimated that lung cancer accounts for approximately 2 million new cases and an overwhelming 1.76 million deaths each year [2]. The ubiquity and lethality of lung cancer transcend geographical and socio-economic boundaries, positioning it as a universal challenge that demands a concerted and multi-faceted response from the global health community.

The burden of lung cancer is not merely a reflection of its high incidence and mortality rates but also highlights the intricate complexities associated with its diagnosis, treatment, and management [3]. Lung cancer presents in various histological subtypes, each with its own distinct molecular profile and clinical trajectory, further complicating the landscape of care [4, 5]. Moreover, the majority of lung cancer cases are diagnosed at an advanced stage, when the prospects for curative treatment are significantly diminished [6]. This late-stage diagnosis is partly attributed to the subtle and non-specific nature of early symptoms, as well as the limitations in current screening and diagnostic methodologies [7]. The risk factors for lung cancer are well documented, with tobacco smoking being the most prominent [8]. However, a significant proportion of cases occur in non-smokers, implicating a range of other etiological factors, including environmental and occupational exposures, genetic predispositions, and lifestyle elements [9]. Therefore, the multifactorial nature of lung cancer etiology underscores the need for a comprehensive understanding of its risk factors and pathogenesis to devise effective prevention and early detection strategies.

In the face of these challenges, the past few decades have witnessed remarkable advancements in the field of lung cancer research and treatment. Currently, lung cancer patients receive surgery, chemotherapy, radiotherapy, and targeted therapy as treatments [10]. Among them, the advent of targeted therapy and immunotherapy has revolutionized the therapeutic landscape, offering new hope to patients, especially those with advanced disease [11]. These advancements have been propelled by a deeper understanding of the molecular mechanisms underlying lung cancer, including the pivotal role of genetic alterations. In particular, mutations in the TP53 gene, which plays a critical role in cellular processes such as DNA repair, apoptosis, and cell cycle regulation, have been identified in a significant proportion of lung cancer cases [12, 13]. The TP53 pathway's involvement in tumorigenesis and cancer progression makes it a critical target for therapeutic intervention and a valuable biomarker for disease prognostication and treatment response prediction [14-16]. In addition to its oncogenic role, TP53 expression levels also have significant implications for therapeutic outcomes. Studies have shown that TP53 mutations and altered expression influence the sensitivity of tumors to various treatment strategies, including chemotherapy, targeted therapy, and immunotherapy. For instance, TP53 mutations have been associated with resistance to chemotherapy and radiotherapy, as the loss of TP53 function impairs the DNA damage response and apoptotic pathways [17, 18]. Furthermore, the expression of TP53 has been shown to impact the immune microenvironment, influencing the effectiveness of immunotherapy [19]. In this context, understanding TP53 expression levels in tumors is crucial for predicting treatment response and guiding the selection of appropriate therapeutic approaches. Given the substantial burden of lung cancer and the central role of genetic and molecular factors in its pathogenesis and treatment response, there is a pressing need for continued research into the development of novel diagnostic, prognostic, and therapeutic strategies [20].

In this context, the exploration of TP53-associated transcriptomic signatures represents a promising frontier. Such signatures have the potential to refine the prognostication of lung cancer outcomes and predict responses to emerging treatment modalities, including immunotherapies.

## 2 METHODS

### 2.1 Data Source

The gene expression profiles used for the identification of TP53-associated genes in NSCLC were obtained from the publicly available GEO dataset (https://www.ncbi.nlm.nih.gov/geo/; GSE8569, n = 69), which contains microarray-based transcriptomic data derived from surgically resected lung tumor tissues. A total of 69 NSCLC tumor samples with high-quality RNA were selected, including 41 TP53-mutant (TP53+) and 28 TP53-wildtype (TP53−) cases. These tissue samples were provided by the CNIO Tumor Bank Network in collaboration with multiple Spanish hospitals. Pathological classification was performed independently by two certified pathologists according to the 2004 WHO classification [21]. The RNA sequencing data and the corresponding clinical data of lung cancer samples were deposited in the TCGA database (https://portal.gdc.cancer.gov/). All data related to the study were gathered from the TCGA and GEO databases. The acquisition and application of data were conducted following TCGA and GEO publication guidelines and data access policies. Duplicate genes were assigned the average expression value in this study. The expression data were normalized by log2(value+1) transformation [22]. A549 and H1299 cells were provided by the Nanjing University of Chinese Medicine (Nanjing, China).

### 2.2 Definition of TP53 Mutation Status

To distinguish between TP53-mutant (TP53+) and TP53-wildtype (TP53−) samples, we adopted the classification criteria provided in the original GSE8569 study. Specifically, TP53 mutation status was determined by sequencing exons 5–8 of the TP53 gene, which represent the most frequently mutated regions in non-small cell lung cancer. The mutation screening was conducted using PCR amplification and direct automatic sequencing following standard protocols, as described in the original dataset references [21]. Samples with any non-synonymous mutation in these exons were classified as TP53-mutant (TP53+), while those without such mutations were classified as TP53-wildtype (TP53−).

### 2.3 Screening of TP53-associated Genes in Lung Cancer

A total of 69 NSCLC tumor samples (N_TP53+_ = 41, N_TP53-_ = 28) were analyzed to screen TP53-associated differentially expressed genes in lung cancer. The R package “limma” was utilized to ascertain TP53-associated differentially expressed genes (DEGs) between TP53+ and TP53- groups. The threshold was set as a *p*-value < 0.05 and | log2 fold change (FC)| > 0.5 [23].

### 2.4 Functional Analyses of TP53-associated Differentially Expressed Genes

The R packages “clusterProfiler” and “org.Hs.eg.db” were utilized to perform the gene and Encyclopedia of Genome (KEGG) pathway and gene ontology (GO) enrichment analysis. The threshold was set as a *p*-value < 0.05. The R packages “ggplot2”, “igraph” and “ggraph” were utilized to visualize the results. We used the Search Tool for the Retrieval of Interaction Genes (STRING, https://www.string-db.org/) to explore the potential interaction between two different genes with a confidence value ≥ 0.4 (intermediate) [24]. The protein-protein networks were visualized using the R packages “igraph” and “ggraph”.

### 2.5 Screening of Differentially Expressed Genes and Prognostic Genes in Lung Cancer from TCGA

A total of 1041 lung cancer tumor samples and 108 paracancerous samples from TCGA database were analyzed to screen differentially expressed genes and prognostic genes in lung cancer from TCGA. The R package DESeq2 was utilized to ascertain differentially expressed genes, and the R package “survival” was utilized to ascertain prognostic genes using the Cox proportional hazards method [25]. The high-risk group and low-risk groups of lung cancer were divided by 1-50 and 51-100. The threshold was set as a *p*-value < 0.05.

### 2.6 Construction of Transcriptomic Signature for Survival Prognostication

Our study took the intersection of the TP53-associated genes, differentially expressed genes, and prognostic genes as the potential genes for the transcriptomic signature for survival prognostication. The R package “glmnet” was utilized to obtain valuable genes with the least absolute shrinkage and selection operator (LASSO) analysis. The LASSO algorithm addresses collinearity and overfitting by excluding genes that may have high correlations with other genes [26]. The key genes were then analyzed by univariate and multivariate Cox models. Finally, a prognostic risk score model was built through multivariate Cox regression [27]. The risk score values of patients were calculated by the expression levels and correlation coefficients of their transcriptomic signatures. The nomogram, according to the multivariate Cox regression analysis results, was developed using the R package “rms”. The ROC curves and survival analysis were utilized to evaluate the predictive value of the transcriptomic signature between low- and high-risk score groups [28].

### 2.7 The Immune Landscape of the Transcriptomic Signature

Single-sample gene set enrichment analysis (ssGSEA) was utilized to apply genetic markers to each cancer sample based on immune cell populations and immune pathways, for quantifying the infiltration score of 16 immune cells and the activity of 13 immune-related pathways, using the R package “gsva” [29]. The immune landscape of high- and low-expression-level groups of a single gene from the transcriptomic signature was investigated. Additionally, the immune landscape of high- and low-risk groups of transcriptomic signatures was also investigated by ssGSEA, estimate and CIBERSORT methods.

### 2.8 The Expression Level of Target Genes Through qPCR

A549 cells were cultured in complete medium (RPMI-1640 supplemented with 10% FBS and 100 U/mL penicillin-streptomycin). H1299 cells were cultured in complete medium (DMEM/F12 supplemented with 10% FBS and 100 U/mL penicillin-streptomycin). All cells were incubated at 37°C with 5% CO_2_ for 24 hours. A549 cells have been characterized to express wild-type p53, while H1299 cells are reported to be p53-null (p53.free.fr/Database/Cancer_cell_ lines/HB_cell_lines.html). Total RNA was extracted from the samples using an RNA isolation kit, as per the manufacturer's instructions. Subsequently, cDNA synthesis was performed using the cDNA synthesis kit to serve as template DNA for real-time qPCR assays. RT-qPCR assays were conducted on the target genes using Green qPCR SuperMix on a real-time detector. Each experiment was repeated on three separate occasions in order to obtain the mean and standard error of the measurement (SEM). Statistical analysis was conducted using Graphpad Prism 9.5.1, and *p*-values were calculated (*p<0.05 and **p<0.01). The specific details of the reagents and primers utilized for the genes can be found in Supplementary Information **S1**.

## 3 RESULTS

 The aim of this study was to identify DEGs between TP53-mutated (TP53+) and TP53-wildtype (TP53-) NSCLC tumor samples and to develop a gene signature that can predict overall survival and immune response, particularly in the context of immunotherapy.

### 3.1 TP53-associated Genes in Lung Cancer

As shown in Fig. (**1A**), a total of 535 DEGs were identified between TP53+ and TP53- groups, consisting of 168 up-regulated genes and 367 down-regulated genes. The expression levels of these genes in tumor samples are shown in the heatmap in Fig. (**1B**). As shown in Fig. (**1C**), KEGG and GO analysis results show that these DEGs are mainly concentrated in cellular functions, immune system mechanisms, and responses to bacterial infections, which include regulation of cell-cell adhesion, leukocyte-mediated immunity, positive regulation of leukocyte activation, the external side of the plasma membrane, the immunological synapse, the lamellar body, serine-type endopeptidase activity, growth factor binding, fibroblast growth factor binding, cell adhesion molecules, complement and coagulation cascades, and Staphylococcus aureus infection. The protein-protein interaction network was built and visualized in Fig. (**1D**), consisting of 183 nodes and 843 edges.

### 3.2 Transcriptomic Signature for Survival Prognostication

 After screening differentially expressed genes and prognostic genes in lung cancer, a total of 32521 genes were identified as DEGs and 3287 genes as prognostic genes. After intersecting with TP53-associated differentially expressed genes, 29 genes were included in further LASSO analysis for the construction of the transcriptomic signature for survival prognostication as shown in Fig. (**2A**). As shown in Figs. (**2B** and **2C**), through determining the best lambda value by 10-fold cross-validation, a total of 12 genes, including HMGA1, ANLN, BARX2, BMP2, EREG, IER3, KRT8, LPXN, PRC1, TNNT1, OGT and SNX30, were utilized to construct the transcriptomic signature (Table **S1**). The risk score was computed as follows: risk score = 0.009*HMGA1 + 0.063*ANLN + 0.031*BARX2 + 0.065*BMP2 + 0.047*EREG + 0.049*IER3 + 0.039*KRT8 - 0.027*LPXN + 0.235*PRC1 + 0.007*TNNT1 - 0.118*OGT - 0.031*SNX30. Moreover, we conducted univariate and multivariate Cox analyses on these 12 genes. As shown in (Fig. **2D**), the univariate Cox analysis results showed that 10 genes had significant prognostic value: HMGA1(1.165 (1.066 - 1.273), Pval< 0.001), ANLN(1.167 (1.078 - 1.262), Pval< 0.001), BARX2(1.086 (1.027 - 1.148), Pval=0.004), BMP2(1.090 (1.014 - 1.172), Pval=0.019), EREG(1.078 (1.030 - 1.127), Pval=0.001), IER3(1.158 (1.073 - 1.251), Pval< 0.001), KRT8(1.098 (1.004 - 1.201), Pval=0.040), TNNT1(1.062 (1.015 - 1.112), Pval=0.009), OGT(0.819 (0.726 - 0.923), Pval=0.001), SNX30(0.878 (0.794 - 0.971), Pval=0.011) (Table **S2**). As shown in (Fig. **2E**), in the multivariate Cox analysis results, three of these genes still exhibited significant prognostic significance, BMP2(1.115 (1.029 - 1.208), Pval=0.008), EREG(1.069 (1.013 - 1.128), Pval=0.016), OGT(0.841 (0.725 - 0.976), Pval=0.023) (Table **S2**). Fig. (**2F**) illustrates the nomogram, which integrates the expression levels of 12 selected genes along with age, gender, and pathological stage to estimate the 1-, 3-, and 5-year survival probabilities for NSCLC patients. Each variable is assigned a point on the uppermost scale, and the total score is mapped to a survival probability scale, providing individualized prognostic assessment. Fig. (**2G**) presents the calibration plot, assessing the agreement between the predicted survival probabilities from the nomogram and the actual observed outcomes at 1, 3, and 5 years. The close alignment of the calibration curves with the 45-degree ideal line indicates that the nomogram has strong predictive accuracy.

### 3.3 Lung Cancer Survival Rates

We observed the time-dependent AUC curve of the transcriptomic signature (Fig. **3A**). The data presented in Fig. **3** were obtained from the TCGA database (https://portal.gdc.cancer.gov/), specifically from the TCGA-LUAD and TCGA-LUSC cohorts. A total of 1149 RNA sequencing (RNA-seq) profiles from lung cancer tumor tissues and 108 adjacent normal lung tissues were included in the analysis. The RNA-seq data were processed using the STAR aligner and quantified in transcripts per million (TPM) format, ensuring accurate and comparable expression measurements across samples. The gene expression profiles were used to compare the expression levels of the selected prognostic genes between tumor and normal tissues, and between different risk groups as defined by the prognostic model. Lung cancer patients were then divided into low- and high-risk prognosis groups using the survival time-dependent ROC. The survival analysis illustrated that compared with the high-risk score group, the low-risk score group had a better outcome (HR = 1.774(1.449 - 2.172), *P* 0.001) (Fig. **3B**). The ROC curve shows that the 1-, 2- and 3-year overall survival (OS) predicted AUC of TIPM are 0.629, 0.631 and 0.601, respectively (Fig. **3C**). As shown in Fig. (**3D**), the low- and high-risk prognosis groups differ clearly depending on their status (0 or 1). The gene HMGA1 carries the most weight among the gene-related factors, as indicated by its scale reaching the highest point count. Other genes, such as SNX30 and LPXN, also contribute significantly, suggesting their influential role in the model. Meanwhile, genes like PRC1 and KRT8 are assigned fewer points, reflecting a potentially lower impact on the survival outcome. Age and gender, the two demographic variables included in the model, are depicted as binary categorical factors. Gender differences are marked, with female patients being assigned a baseline reference value and male patients receiving additional points, implying a distinction in survival outcomes based on gender. Age is divided at a threshold of 65 years, with those older than 65 being allocated additional points, indicating age as a factor in survival probability. Pathologic stages of lung cancer, ranging from Stage I to Stage IV, are factored into the model as categorical variables. The stage contributes significantly to the total points, emphasizing the clinical importance of the cancer's extent in determining survival.

### 3.4 The Immune Landscape of the Transcriptomic Signature

As shown in Figs. (**4A** and **S1**), each gene in the transcriptomic signature is closely related to the immune pathways of various immune cells associated with immune infiltration. For the low and high-risk groups defined by the risk score, ssGSEA analysis reveals that several immune cells, such as aDC (activated Dendritic Cells), CD8 T cells, Eosinophils, Neutrophils, NK CD56dim cells, T cells, Tcm (Central Memory T cells), Tem (Effector Memory T cells), TFH (T Follicular Helper cells) are significantly different between groups (Fig. **4B**). The estimate analysis shows that the high-risk group has a higher ImmuneScore and ESTIMATEScore (Fig. **4C**). Furthermore, the CIBERSORT analysis shows that many cells, including Plasma cells, T cells CD4 memory resting, Monocytes, Macrophages M0, Macrophages M1, Macrophages M2, Dendritic cells activated, and resting mast cells, have a significant difference in enrichment scores (Fig. **4D**). The results suggest that the transcriptomic signature can predict the situation of immune infiltration to a certain degree.

### 3.5 Expression of Target Genes on Different NSCLC Cells

Expression levels of the 12 selected genes were evaluated by qPCR to compare the TP53+ (A549 cell) and TP53- (H1299 cell) groups. As shown in (Fig. **5**), relative to the TP53- group, the TP53+ group exhibited significantly decreased expression of BMP2, LPXN, OGT, and SNX30, and significantly increased expression of IER3, ANLN, TNNT1, KRT8, BARX2, and PRC1.

These findings show that the 12-gene signature effectively stratifies patients into low- and high-risk groups, providing prognostic insight and identifying genes that may guide future immunotherapy strategies. The nomogram integrates the expression levels of the selected genes along with clinical variables such as age, gender, and stage, to predict survival. The Kaplan–Meier survival analysis further validates the model's ability to distinguish between high-risk and low-risk groups.

## 4 DISCUSSION

The study first presents an analysis of TP53-associated differentially expressed genes in lung cancer, culminating in the construction of a transcriptomic signature with significant prognostic value. TP53, a gene frequently mutated in various forms of cancer, plays a pivotal role in cell cycle regulation and apoptosis [30]. In lung cancer, the status of TP53 is a critical determinant of the tumor's behavior and patient outcome [31]. By integrating data from public databases such as GEO and TCGA, this work not only identifies a set of DEGs linked to TP53 status but also correlates these with patient survival and the potential response to immunotherapy. Through a series of analyses, including differential expression analysis, pathway enrichment, and protein-protein interaction networks, followed by LASSO and Cox regression modeling, our study develops a reliable 12-gene prognostic signature. This signature not only stratifies lung cancer patients into distinct risk categories but also provides insight into the underlying immune response, offering dual utility in both prognostication and therapeutic guidance.

In this study, we identified a 12-gene transcriptomic signature associated with TP53 status that provides prognostic insights for NSCLC patients. The link between this signature and clinical outcomes, particularly overall survival, is evident from the Kaplan–Meier survival analysis, which demonstrated a significant difference in survival between patients stratified into low- and high-risk groups based on their transcriptomic risk scores. However, it is important to further investigate the specific roles of these 12 genes in a clinical context. The 12 selected genes are involved in various biological pathways, such as cell cycle regulation, immune modulation, and cell adhesion, which are crucial for tumor progression and response to therapies. The prognostic significance of these genes suggests that they may impact tumor behavior through their roles in these pathways, influencing the patient’s clinical outcome. Future studies are needed to explore how the expression levels of these genes correlate with treatment response, particularly to immunotherapy, and how they may be integrated into clinical decision-making.

The 12-gene signature comprises HMGA1, ANLN, BARX2, BMP2, EREG, IER3, KRT8, LPXN, PRC1, TNNT1, OGT, and SNX30, representing diverse biological processes and molecular pathways implicated in lung cancer pathogenesis. Each gene has been independently associated with cancer-related processes in previous studies, strengthening the biological validity of our signature. These genes are involved in critical cellular processes that are frequently dysregulated upon TP53 mutation, providing mechanistic insights into how TP53 status influences lung cancer progression and immune response. HMGA1 (High-mobility group AT-hook 1) functions as a chromatin architectural factor that exhibits altered expression patterns in TP53-mutant tumors [32, 33]. In NSCLC, HMGA1 promotes metastasis through pathways that are normally suppressed by wild-type TP53, representing a critical downstream effector of TP53 dysfunction [34, 35]. ANLN (Anillin actin-binding protein), essential for cytokinesis and cell cycle progression, shows elevated expression in TP53-mutant tumors due to loss of TP53-mediated cell cycle checkpoint controls [36-38]. This TP53-dependent dysregulation of ANLN contributes to chromosomal instability and accelerated tumor growth in NSCLC [39, 40]. BARX2 (BarH-like homeobox 2) exhibits altered regulation in the context of TP53 mutation, affecting cellular adhesion and cytoskeletal organization [41, 42]. In NSCLC, BARX2 suppresses tumor progression through inhibition of the Wnt/β-catenin pathway, a function that may be compromised in TP53-mutant tumors [43-45]. BMP2 (Bone Morphogenetic Protein 2), a member of the TGF-β superfamily [46], shows differential expression between TP53-mutant and wild-type tumors, influencing the tumor microenvironment and immune cell recruitment [47-51]. In NSCLC, BMP2 overexpression correlates with advanced disease and metastasis, particularly in TP53-mutant contexts [52, 53]. EREG (Epiregulin), an EGF family member, exhibits altered expression in TP53-mutant tumors, affecting epithelial-mesenchymal transition (EMT) and immune evasion mechanisms [54, 55]. In NSCLC, EREG contributes to therapeutic resistance [56-58], a hallmark of TP53-mutant tumors. PRC1 (Protein Regulator of Cytokinesis 1), involved in cell division regulation, shows dysregulated expression in the absence of functional TP53 [59, 60]. In lung adenocarcinoma, PRC1 activation promotes aggressive disease progression [61, 62], particularly in tumors with compromised TP53 function. KRT8 (Keratin 8) expression patterns differ between TP53-mutant and wild-type tumors, affecting epithelial integrity and immune cell interactions [63-65]. In NSCLC, KRT8 enhances TGF-β signaling and promotes EMT [66], processes that are modulated by TP53 status [67]. TNNT1 (Troponin T1) shows differential expression based on TP53 status and influences cellular migration patterns that affect immune cell infiltration [68-70]. IER3 (Immediate Early Response 3) participates in TP53-dependent stress response pathways [71, 72], with altered function in lung tumors contributing to sustained ERK activation [73]. LPXN (Leupaxin) mediates cell adhesion processes that are disrupted in TP53-mutant tumors, affecting tumor-immune cell interactions [74-77]. OGT (O-GlcNAc transferase) catalyzes modifications that influence TP53 function and stability [78], while SNX30 (Sorting Nexin 30) participates in trafficking pathways that are remodeled in the context of TP53 mutation [79].

Our analyses revealed significant associations between this TP53-associated gene signature and immune cell infiltration patterns, with the high-risk group exhibiting distinct immune profiles. These findings establish a direct link between TP53 mutational status, transcriptional dysregulation, and alterations in the tumor immune microenvironment, offering valuable insights for immunotherapy stratification in NSCLC patients. Furthermore, TP53 mutations have been shown to impair the expression of key genes involved in antigen presentation (*e.g*., MHC class I molecules) and immune surveillance, thereby contributing to immune escape [80, 81]. For instance, loss of TP53 function is associated with reduced interferon signaling and impaired recruitment of cytotoxic T cells, which are critical for effective anti-tumor immunity [82, 83]. Several genes in our 12-gene signature, including HMGA1, KRT8, and EREG, have been implicated in modulating immune checkpoints, epithelial–mesenchymal transition (EMT), and cytokine secretion, all of which influence the tumor immune microenvironment and potentially attenuate responses to immunotherapy [84-88]. The high-risk group, characterized by upregulation of these genes also displayed lower levels of activated CD8+ T cells and higher infiltration of immunosuppressive cells such as Tregs and M2 macrophages, suggesting a more immune-evasive phenotype. These observations provide a plausible biological explanation for the poorer therapeutic outcomes often observed in TP53-mutant NSCLC patients and underscore the importance of integrating TP53 mutational status and immune gene signatures when designing personalized immunotherapeutic strategies.

Several limitations should be acknowledged. First, the prognostic gene signature was developed and validated using retrospective datasets (GEO and TCGA), and no independent clinical cohort was included for external validation. Second, although we explored immune cell infiltration based on transcriptomic data, functional validation of the immune-modulatory roles of the identified genes is still lacking. Third, the *in vitro* qPCR validation was limited to two lung cancer cell lines (A549 and H1299), which may not fully capture the heterogeneity of NSCLC in patients. Additionally, the prognostic signature was derived primarily from TP53 mutation status, which may not account for other genomic or epigenetic alterations influencing patient prognosis. Future studies should aim to validate the prognostic signature in large, multicenter, and prospective clinical cohorts. Moreover, functional experiments are needed to elucidate the biological mechanisms by which these genes influence tumor progression and the immune microenvironment. Integrating multi-omics data and investigating the response to specific immunotherapies in TP53-stratified patients may further enhance the clinical utility of this signature.

## CONCLUSION

In summary, this study systematically identified TP53-associated differentially expressed genes in non-small cell lung cancer (NSCLC) using an integrated analysis of GEO and TCGA datasets. Through LASSO regression and Cox proportional hazards modeling, we developed a robust 12-gene transcriptomic signature that effectively stratifies lung cancer patients into distinct prognostic groups. This signature not only demonstrated predictive value for overall survival but also revealed significant associations with immune cell infiltration and immune pathway activity, suggesting its potential as a biomarker for both prognosis and immune landscape assessment. Moreover, qPCR validation in TP53+ (A549) and TP53− (H1299) lung cancer cell lines confirmed the differential expression of key genes within the signature, supporting its biological relevance. However, this study is limited by the lack of prospective clinical validation and functional experiments. Future research should focus on validating the signature in larger, independent cohorts and elucidating the mechanistic roles of these genes in tumor progression and immune regulation. Overall, our findings provide valuable insights into the transcriptomic alterations associated with TP53 mutation status in lung cancer and offer a promising gene signature for risk stratification and future immunotherapy guidance.

## Figures and Tables

**Fig. (1):**
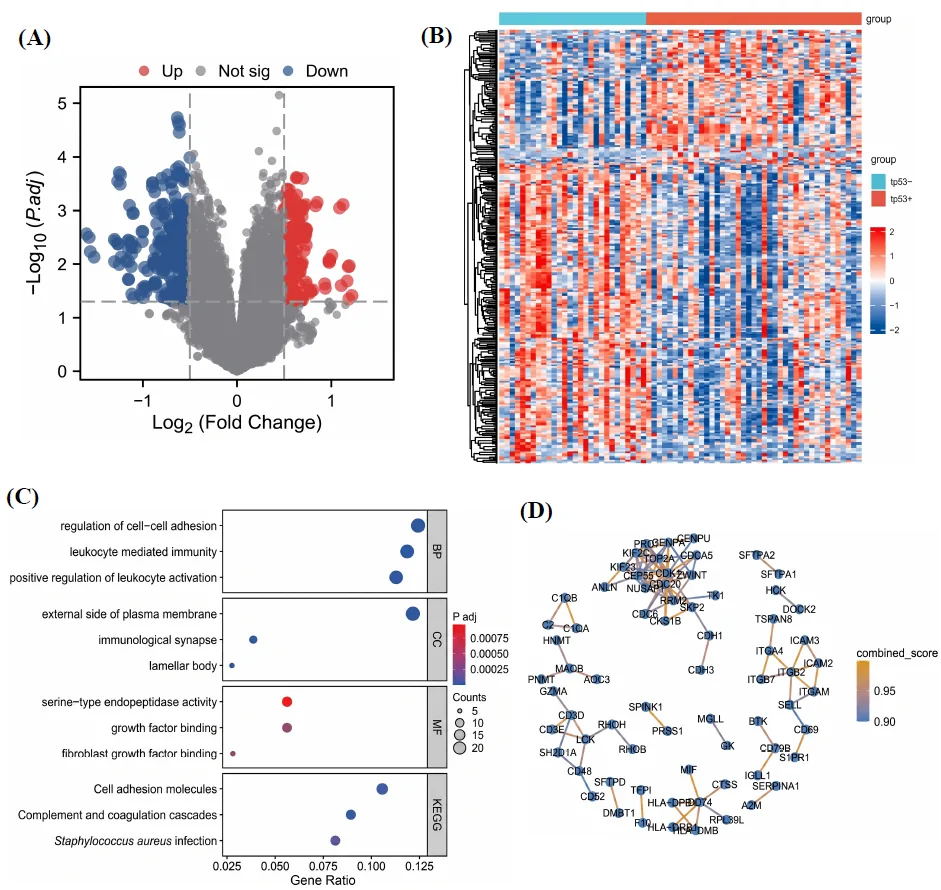
Identification and functional characterization of DEGs between TP53+ and TP53- Lung Cancer Phenotypes. (**A**) The volcano plot of the DEGs between TP53- and TP53+ patients of lung cancer. (**B**) The heatmap of the DEGs between TP53- and TP53+ patients of lung cancer. (**C**) The KEGG and GO enrichments of the DEGs between TP53- and TP53+ patients of lung cancer. (**D**) The PPI network of the DEGs between TP53- and TP53+ patients of lung cancer. The data set contains 69 non-small cell lung cancer tumor samples (N_TP53+_ = 41, N_TP53-_ = 28).

**Fig. (2):**
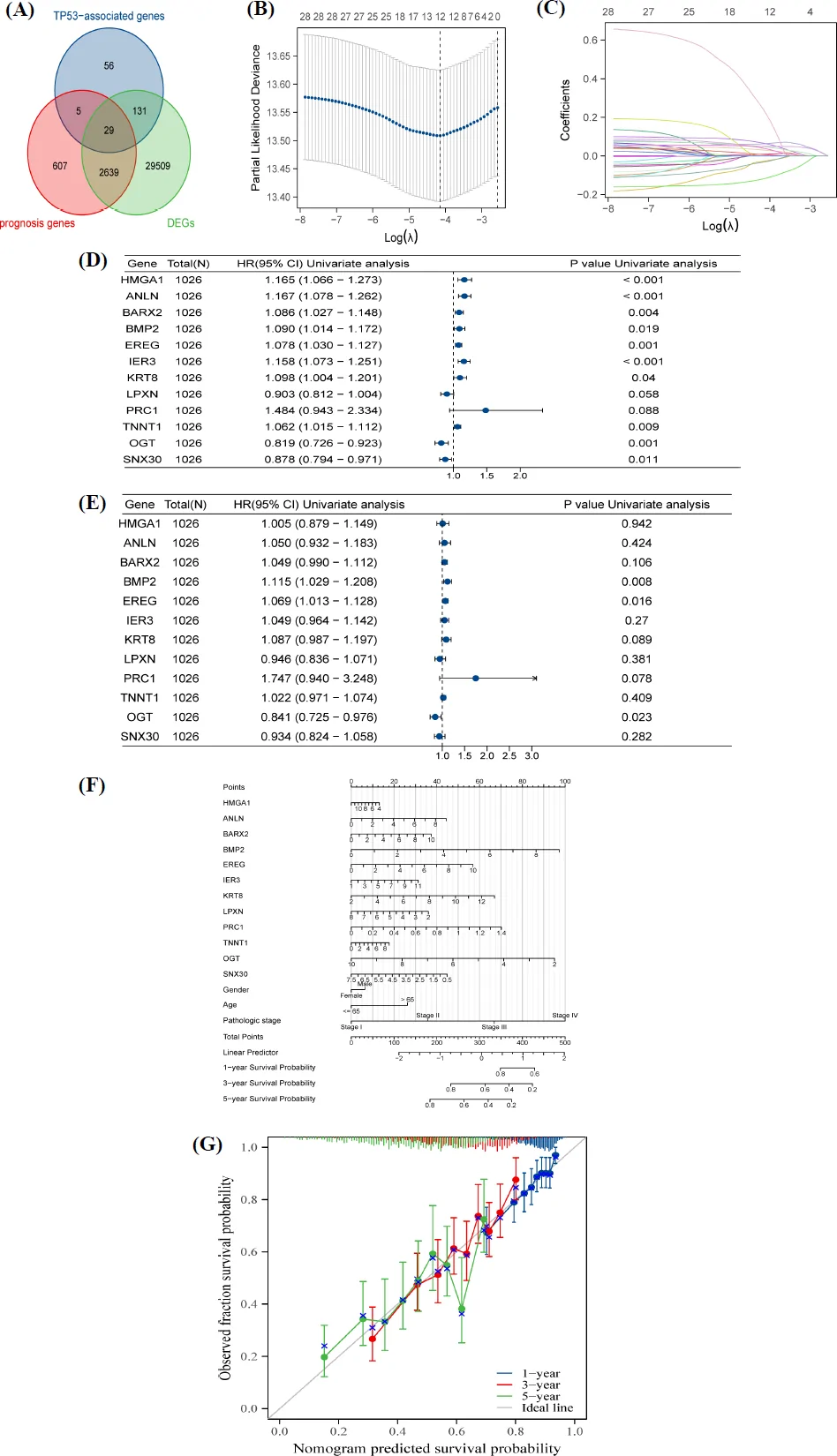
Development and validation of a 12-gene TP53-associated transcriptomic signature for survival prediction in NSCLC. (**A**) The interaction of TP53 associated DEGs, lung cancer associated DEGs and prognostic genes. (**B**) LASSO regression with 10-fold cross-validation identified 29 hub genes. (**C**) LASSO coefficient profiles of 29 genes. The forest diagrams display the univariate (**D**) and multivariate (**E**) analysis. (**F**, **G**) The prognostic nomogram predicting 1-, 3- and 5-year overall survival of lung cancer.

**Fig. (3):**
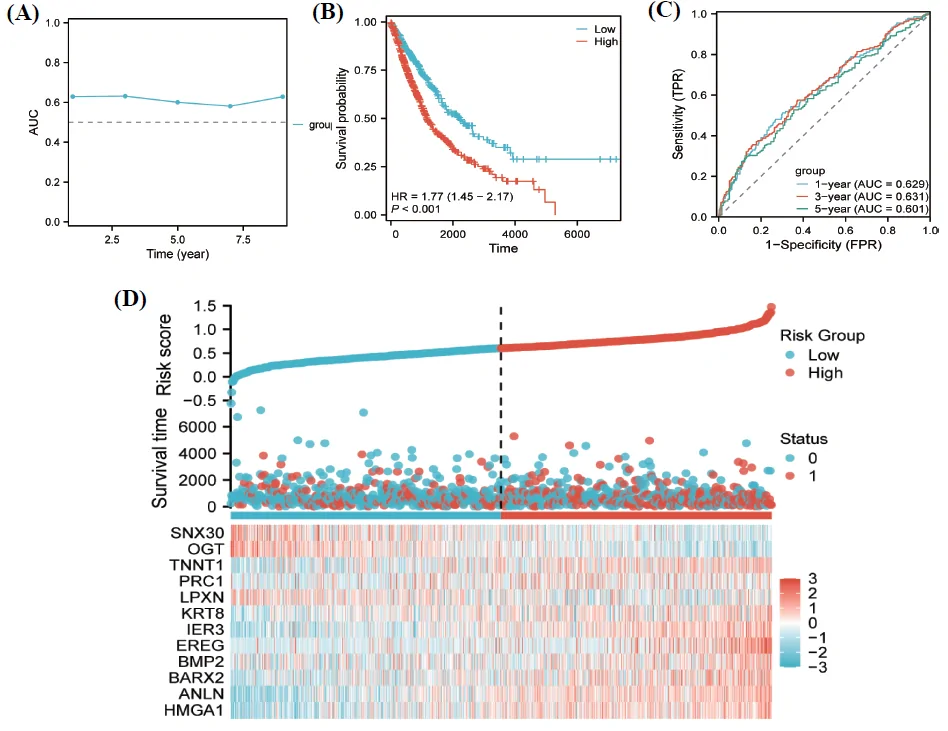
Validation and performance assessment of the 12-gene TP53-associated prognostic signature in TCGA lung cancer cohorts. (**A**) The time-dependent AUC curve of the transcriptomic signature. (**B**) The Kaplan-Meier survival analysis of low and high-risk groups. (**C**) The ROC curve of low and high-risk groups predicting 1-, 3- and 5-year overall survival of lung cancer. (**D**) The gene expression of the transcriptomic signature between low and high-risk groups.

**Fig. (4):**
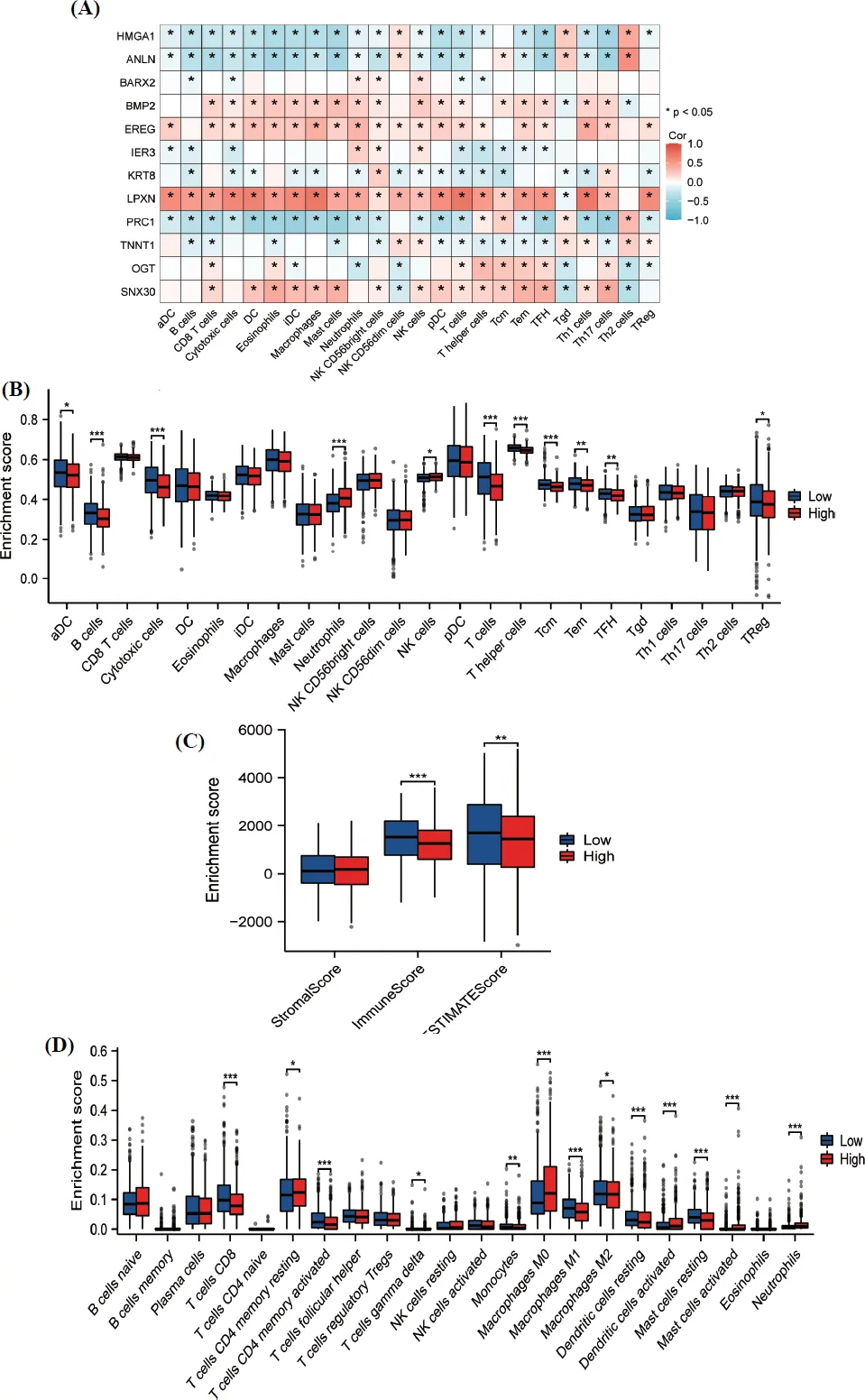
Immunological profile analysis of the TP53-ssociated 12-gene. (**A**) The relationships between the genes of the transcriptomic signature and immune infiltration. (**B**) The immune landscape of low and high-risk groups using ssGSEA analysis. (**C**) The enrichment score of immune infiltration using ESTIMATE analysis. (**D**) The immune landscape of low and high-risk groups using CIBERSORT analysis. Results are means ± SEM. *p0.05, **p0.01, and ***p0.001.

**Fig. (5):**
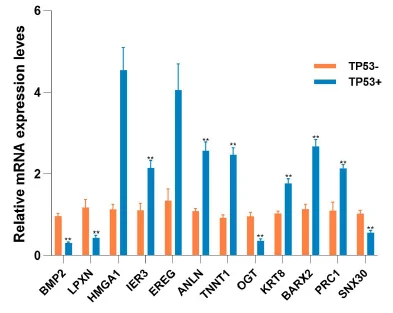
The expression of 12 selected genes mRNA level. Results are means ± SEM. **p0.01.

## Data Availability

The authors confirm that the data supporting the findings of this research are available within the article.

## LIST OF ABBREVIATIONS

Anln: Anillin Actin-Binding Protein
Auc: Area Under the Curve
Barx2: Barh-Like Homeobox 2
Bmp2: Bone Morphogenetic Protein 2
Degs: Differentially Expressed Genes
Emt: Epithelial-Mesenchymal Transition
Ereg: Epiregulin
Go: Gene Ontology
Hmga1: High-Mobility Group At-Hook 1
Ier3: Immediate Early Response 3
Krt8: Keratin 8
Lasso: Least Absolute Shrinkage and Selection Operator
Lpxn: Leupaxin
Nsclc: Non-Small Cell Lung Cancer
Ogt: O-Glcnac Transferase
Prc1: Protein Regulator Of Cytokinesis 1
Qpcr: Quantitative Real-Time PCR
Snx30: Sorting Nexin 30
Ssgsea: Single-Sample Gene Set Enrichment Analysis
Tnnt1: Troponin T1
Tpm: Transcripts Per Million
