# Supplementary material for: Transcriptomic Signatures in TP53 Positive and Negative Tumor Samples in NSCLC
Source: Curr Gene Ther. 2025 Aug 8;26(5):E15665232357300. doi: 10.2174/0115665232357300250805021330 (PMC13625031; doi:10.2174/0115665232357300250805021330)
Supplement: Supplementary file 1 [file CGT-26-5-E15665232357300_SD1.pdf]

Supplementary Material

Transcriptomic Signatures in TP53 Positive and Negative Tumor Samples in NSCLC

Miao Xie[1], Baoguang Liu[2], Ziyi Chen[1], Tongtong Cao[1] and Xiaoyan Zhang[1,*]

[1]*College of Traditional Chinese Medicine, Henan University of Chinese Medicine, Zhengzhou, Henan, China;* [2]*Department of Chinese Medical Sciences, Henan University of Chinese Medicine, Zhengzhou, Henan, China*

1. The primers for the target genes

Gene	Forward primer	Reverse primer
BMP2	GGAACGGACATTCGGTCCTT	CACCATGGTCGACCTTTAGGA
LPXN	CCACCACCTTCTAAAACGTCAG	CCCAAGCATTGAGTCCAGGG
HMGA1	GCTGGTAGGGAGTCAGAAGGA	TGGTGGTTTTCCGGGTCTTG
IER3	CAGCCGCAGGGTTCTCTAC	GATCTGGCAGAAGACGATGGT
EREG	CAATCCACGTGTGGCTCAAG	ACCCACTTCACACCTGCAGTA
ANLN	TGCCAGGCGAGAGAATCTTC	CGCTTAGCATGAGTCATAGACCT
TNNT1	TGATCCCGCCAAAGATCCC	TCTTCCGCTGCTCGAAATGTA
OGT	TCCTGATTTGTACTGTGTTCGC	AAGCTACTGCAAAGTTCGGTT
KRT8	CAGAAGTCCTACAAGGTGTCCA	CTCTGGTTGACCGTAACTGCG
BARX2	CCTTCCCTGCGGGCATATC	GGGCTGTTCCGTCTCTGAC
PRC1	ACACTCTGTGCAGCGAGTTAC	TTCGCATCAATTCCACTTGGG
SNX30	CTGTTCGTCGAAGATACCAGGA	TGAAAAACGATCCACAACACCT

Supporting Information of Figure S1

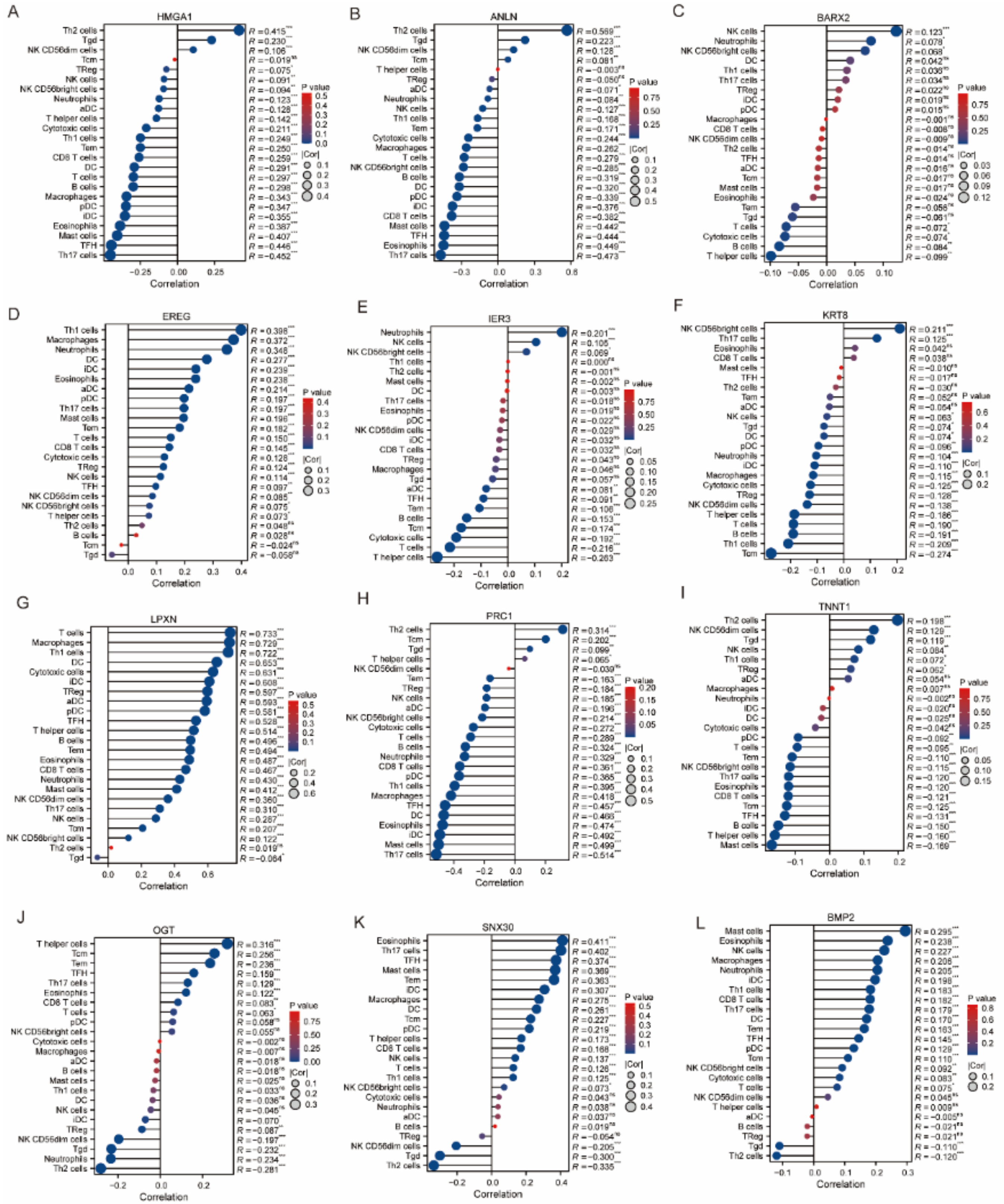

Fig. (S1). Genes in the transcriptome signature correlate with immune infiltration. (**A**)HMGA1. (**B**)ANLN. (**C**)BARX2. (**D**) EREG. (**E**)IRE3. (**F**)KRT8. (**G**)LPXN. (**H**)PRC1. (**I**)TNNT1. (**J**)OGT. (**K**)SNX30. (**L**) BMP2.
